# Output Power Computation and Adaptation Strategy of an Electrosurgery Inverter for Reduced Collateral Tissue Damage

**DOI:** 10.1109/TBME.2022.3225271

**Published:** 2023-05-19

**Authors:** Congbo Bao, Sudip K. Mazumder

**Affiliations:** Electrical and Computer Engineering, University of Illinois Chicago, USA.; Electrical and Computer Engineering, University of Illinois Chicago, Chicago, IL 60607 USA

**Keywords:** Electrosurgery, inverter, power, adaptation, impedance, sensor count, collateral damage

## Abstract

**Objective:**

This paper investigates two ways of output-power computation, namely, sparse- and multi-sampling-based methods, to overcome sampling speed limitation and arcing nonlinearity for electrosurgery. Moreover, an impedance-based power adaptation strategy is explored for reduced collateral tissue damage.

**Methods:**

The efficacy of the proposed power computation and adaptation strategy are experimentally investigated on a gallium-nitride (GaN)-based high-frequency inverter prototype that allows electrosurgery with a 390 kHz output frequency.

**Results:**

The sparse-sampling-based method samples output voltage once and current twice per cycle. The achieved power computing errors over 1000 cycles are 1.43 W, 2.54 W, 4.53 W, and 4.89 W when output power varies between 15 W and 45 W. The multi-sampling-based method requires 28 samples of both outputs, and the corresponding errors are 0.02 W, 0.86 W, 1.86 W, and 3.09 W. The collateral tissue damage gauged by average thermal spread is 0.86 mm, 0.43 mm, 1.11 mm, and 0.36 mm for the impedance-based power adaptation against 1.49 mm for conventional electrosurgery.

**Conclusion:**

Both power-computation approaches break sampling speed limitations and calculate output power with small errors. However, with arcing nonlinearity presence, the multi-sampling-based method yields better accuracy. The impedance-based power adaptation reduces thermal spreads and diminishes sensor count and cost.

**Significance:**

This paper exemplifies two novel power-computation ways using low-end industrial-scale processors for biomedical research involving high-frequency and nonlinearly distorted outputs. Additionally, this work is the first to present the original impedance-based power adaptation strategy for reduced collateral damage and it may motivate further interdisciplinary research towards collateral-damage-less electrosurgery.

## I. Introduction

**Fig. 1.**
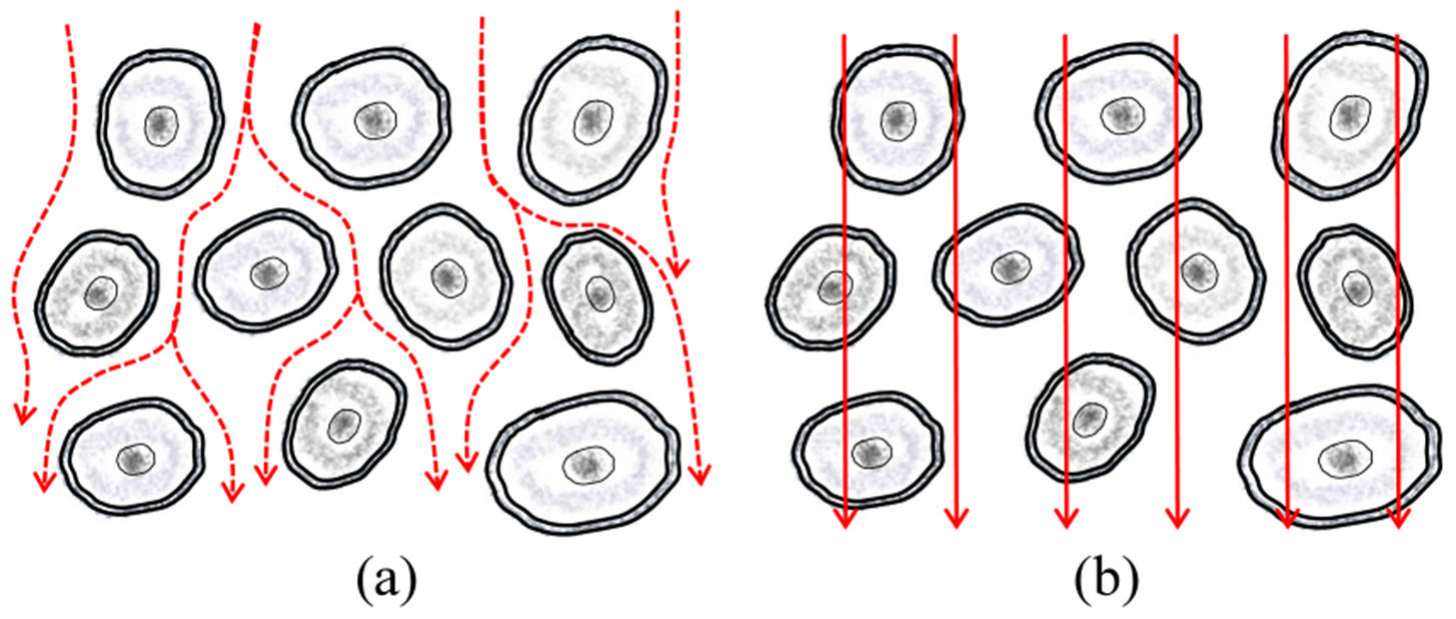
(a) Low- and (b) high-frequency current paths inside the tissue.

BIOMEDICAL tissue is made of numerous cells and cellular membranes introduce capacitance to tissue, therefore, the current path inside the tissue is frequency-dependent [1], [2]. Capacitance blocks DC current and presents a high impedance to low-frequency AC current. Hence, the resultant current path mainly surrounds cells through extracellular liquids, as shown in Fig. 1(a). In contrast, AC current of high frequency passes through both extracellular and intracellular liquid, as pictured in Fig. 1(b). A high-frequency current of sufficient high amplitude, flowing through the cell body, can generate enough Joule heating and raise intracellular liquid temperature to the vaporization point. The concomitant volume expansion of the volatilized liquids gives rise to microscopic cell ruptures, and collectively, produces macroscopical tissue-cutting effects [3]. Besides, muscle and nerve stimulation or pain ends when the applied electrical signal oscillates more than 100 thousand times per second [4]. Grounded on those principles, William Bovie pioneered and devised the first electrosurgery device of real sense about 100 years ago [4]. After that, continuous efforts from researchers promote the development of electrosurgery and enhance the scientific understanding of human society on it. Nowadays, the pursuit of reduced collateral tissue damage becomes one of the critical focuses of modern electrosurgery.

The tissue damage during electrosurgery is tightly related to electrical energy applied to the target tissue. Properly delivered energy not only minimizes additional tissue damage but also shortens the time required for post-surgery recovery. In contrast, inappropriately transmitted energy enhances undesired tissue damage, and increases safety concerns as well. As is well known, electrical energy is the integration of instantaneous power over the period that power from the electrosurgery generator (ESG) is activated. The cutting speed solely determines the time interval spent over a certain length when the electrode moves along the tissue surface trace during electrosurgery. Consequently, both applied power and cutting speed (or time interval of power activated) are theoretically supposed to play an important role in tissue damage during actual electrosurgery. In [5], thermal damage induced by fixed power with different cutting speeds is explored. The experimental results validate the significant impact of cutting speed on electrocuting damage and emphasize the importance of cutting speed control. Moreover, [6], [7] experimentally demonstrate that various activated periods of power lead to different surgical damage even with the same power setting. Instead of varying cutting speed or power activation period, [5], [6], [7], [8], [9] vary applied power and show the important impact of discrete power settings on the overall cutting performance. Beyond that, [3] and [8] further illustrate and elaborate on the generation mechanism of collateral damage due to an ill-suited power setting. Based on the above-mentioned literature, it can be concluded that cutting speed (or power activated interval) and power setting are experimentally proven to be critical for electrosurgery and should be properly controlled to reduce unwanted collateral tissue damage. In actual electrosurgery, the cutting speed or time interval of power activation is at the surgeon’s sole discretion, except that the electrosurgery is autonomously implemented by a robotic arm [10]. In such a case, the cutting speed or power-activated time interval should be controlled by embedded servo motors inside the robotic arm with very high precision and sensitivity. Nonetheless, in either case, both cutting speed and power activation time interval are externally controlled and none of them is regulated by the ESG itself. Meantime, the research topic of this paper centers on the power adaptation of a high-frequency inverter that enables electrosurgical trials, rather than autonomous robotic electrosurgery. Therefore, autonomous control on cutting speed or regulation of power activation time interval is not covered herein.

For conventional electrosurgery, the applied power is exclusively determined and manually entered into ESGs by a surgeon before surgery is initiated. The value of selected power is primarily decided based on surgeons’ cumulative clinical experiences. There is a lack of professional procedures indicating how to quantitatively update the power setting when target tissue changes either due to tissue type or physical property variation, etc. The practical physical and electrical properties of tissue differ when it comes from distinct individuals or even from the same individual and the same tissue type but separate locations, etc. [11]. Under such circumstances, the optimal power choice, ideally speaking, should be so adjusted such that the induced collateral damage is minimized as much as possible. Unfortunately, the power setting is usually maintained the same during conventional electrosurgery until further updates are reimported by surgeons when appreciable undesired electrosurgical effects or collateral damages have been irreversibly generated and observed. Those undesirable effects or additional damage prolong the post-surgery rehabilitation duration and should be avoided as far as possible through timely power adaptation. In practice, it is challenging for surgeons to entirely avoid power setting nonoptimality and thus, added collateral damage because they can hardly precisely and promptly identify tissue property discrepancies or variations. Even if surgeons are hypothetically able to distinguish tissue physical and electrical property fluctuations, it also takes them some time to halt the electrosurgery, manually reload the power setting into ESG and then reinitiate the surgical procedures again. The total time duration for time-sensitive electrosurgery is elongated as the alteration frequency of such processes climbs. The stretched clinical duration increases the risk of clinical failure or might lead to other serious consequences. Given that, there should be a viable tradeoff between the increasing power modification frequency for reduced collateral tissue damage and the decreasing power modification frequency for shortened electrosurgical time consumption.

**Fig. 2.**
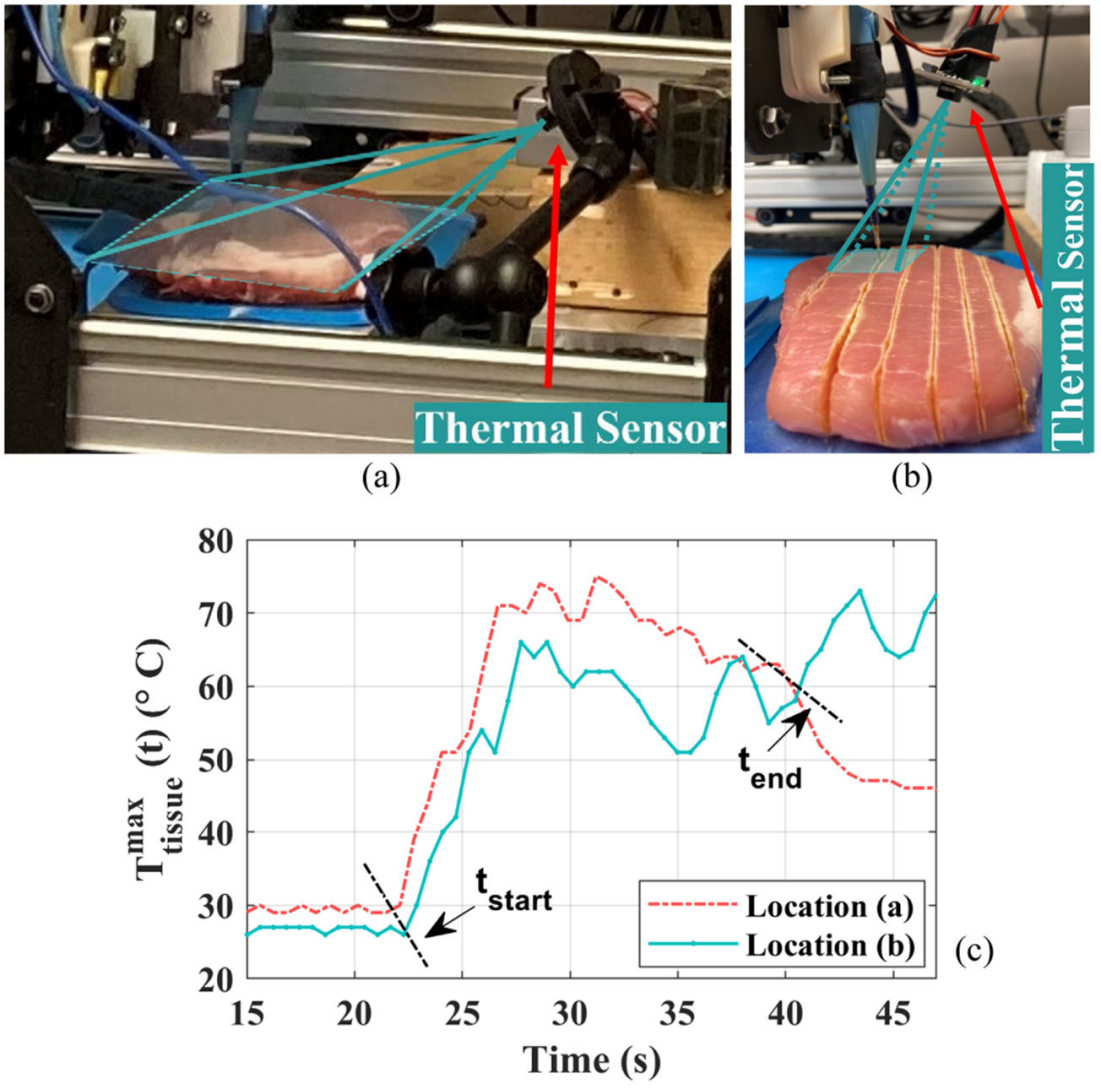
Impact of thermal sensor mounting locations on temperature measurement. The thermal sensor used here is MLX90640 and its field of view during cutting is approximately illustrated as the teal shadow. (a) The sensor is mounted externally to the electrode. (b) The sensor is mounted together with the electrode. (c) The obtained maximum tissue surface temperature. t_start_ and t_end_ indicate the beginning and end of cutting, respectively.

The majority of existing literature work on tracking the manually entered power setting with high accuracy and rapid response [12], [13], [14]. As a result, no real-time power adaptation is adopted in traditional ESG, and surgeons solely take control of the power setting. In contrast to that, a thermal-feedback-based power adaptation that can autonomously modify this power setting is detailed in [8]. Electrocuting traces, conducted on fresh pork using such power adaptation strategy, show superiority over those cut by conventional fixed power in terms of thermal spread and cutting gap uniformity. However, notwithstanding the apparent merits mentioned above, drawbacks exist for the thermal-based power adaptation method. A considerable amount of smoke occurs during electrosurgery [3], and they suffuse between the thermal sensor and tissue, imposing a negative influence on temperature measurement accuracy. Either an advanced and complex thermal sensing compensation algorithm or a costly smoke evacuation pencil is needed to remove the adverse impact of the smoke on tissue surface temperature measurement [15]. Besides, the thermal sensor mounting location also plays an important role in thermal measurement accuracy, as shown in Fig. 2. In Fig. 2(a), the thermal sensor is installed on an external holder such that the entire tissue surface is located within the field view of the sensor. By doing so, the whole tissue surface temperature is measured in each sensing frame, rather than a limited region. In contrast, the thermal sensor in Fig. 2(b) is mounted together with the electrode such that they move together without relative movement during electrocuting. As a result, only the tissue surface temperature, surrounding the electrode tip, is monitored without or with minimal variation of sensing angle and sensing distance between the electrode tip and the thermal sensor. To showcase the temperature measurement difference for two mounting locations, electrocuting trials are conducted on fresh pork under identical experimental conditions. The measured maximum tissue surface temperature is plotted in Fig. 2(c), and significant differences exist between the two sensor-mounting locations, which validates the importance of the thermal installation location. Furthermore, the thermal sensor resolution also matters and imposes an impact on measurement granularity and precision, etc. Higher resolution yields more accurate thermal sensing while, on the other hand, higher resolution also indicates larger thermal data size, heavier data processing burden, and probably higher sensor cost. Finally, it is worth mentioning that the refresh rate of thermal sensors can hardly go beyond 100 Hz. This significantly limits the application of thermal-feedback-based power adaptation in the instance that is highly sensitive to power settings and requires ultrafast power adjustment. Considering all limitations mentioned above of thermal-based power adaptation, the impedance-based ultrafast power adaptation is proposed in Section II-D to serve as one promising substitution. Compared to the conventional constant power scenario, the proposed power adaptation method reduces collateral tissue damage. Meanwhile, it eliminates the limitations linked to the thermal-based tactic with additional benefits of reduced sensor count, shrunken budget and communication requirement, etc. [8]. The efficacy of this novel method is examined on the full-bridge-based high-frequency inverter (HFI) that is initially introduced in [13] with a fundamental (sinusoidal) output frequency of 390 kHz.

## II. Methods

### A. Sparse-Sampling-Based Power Calculation

It is a practical challenge to sample and precisely reconstruct signals of hundreds of kilohertz without a multi-MHz analog-to-digital converter (ADC) sampling rate. Reference [14] samples this kind of high-frequency signal at 50 MHz using Xilinx field-programmable gate array (FPGA), rather than a low-cost industrial-scale digital signal processor (DSP). A high ADC sampling rate is avoided in [12] for average output power computation. However, the output power is indirectly calculated from the input side of the high-frequency inverter with the assumption of a lossless switch network, rather than directly computed from the output (load) side. The practical switches are lossy, especially switched at high frequency. Consequently, the feasibility of this method only works to some extent and needs more examination for diverse situations. Given that the majority of commercial low-end DSPs possess only a few MHz sampling rates, therefore, a power computing algorithm (PCA) is provided to compute the mean value of output power over each cycle. The proposed PCA requires only one output voltage sample and two output current samples per cycle, respectively. These three data can also be utilized to estimate the load impedance in a cycle-by-cycle ultrafast manner.

**Fig. 3.**
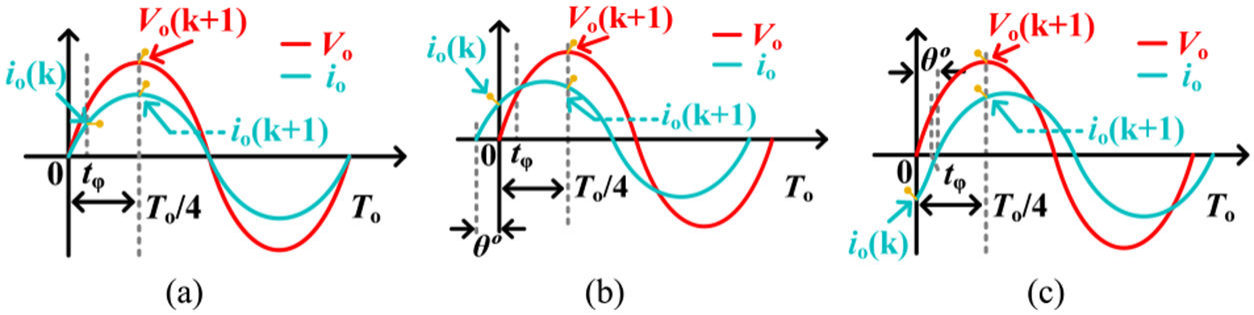
The load characteristics. (a) Output current is in phase with voltage for pure resistive load. (b) Current is leading $\theta^{{}_{{}^{\circ}}}$ for capacitive load. (c) Current is lagging $\theta^{{}_{{}^{\circ}}}$ for inductive load.

When the ideal sinusoidal voltage is applied to a linear load, the resultant current is also sinusoidal and oscillates at the same frequency. The magnitude and phase of the load current depend on load impedance magnitude and type, such as resistive, capacitive, or inductive. Although the Cole-model indicates the capacitive property of the biotissue [16], all three linear load characteristics are delineated in Fig. 3 for the sake of completeness. The procedures to reckon the average output power are formulated as follows:

1. Properly configure the sampling timing of the DSP ADC channel for output voltage $V_{o}(t)$ sensing such that it approximately catches the output voltage positive peak at $T_{s}∕4$, noted down as $V_{o}(k+1)$, which can be easily achieved with HFI in [13]. Although an exact sampling at $T_{s}∕4$ is preferred, there is no need to precisely sample output voltage at $T_{s}∕4$ since sinusoidal signals have minor derivation near the peak. Deviation error is small as long as the sampling point does not seriously deviate from $T_{s}∕4$. Moreover, this kind of error can also be corrected via sensor calibration.
2. Properly configure the DSP ADC current channel such that it samples the output current $i_{o}(t)$ twice per switching cycle. The first sample, denoted as $t_{o}(k)$, initiates at predetermined $t_{\varphi }$ that can be any timing between 0 and $T_{s}∕4$ when taking voltage as the reference. The only requirement for $i_{o}(k)$ is to avoid overlap with the second sample. The second sample is launched at $T_{s}∕4$ and is noted down as $i_{o}(k+1)$. Such a current sampling configuration can also be easily obtained by proper DSP ADC triggering.
3. The quantitative relationship among $i_{o}(k),i_{o}(k+1)$ and output current magnitude $i_{opk}(k)$ is described in (1) if $i_{o}(k)$ is sampled at $t_{\varphi }$ that is equivalent to the electrical angle of φ (in radian). The load impedance angle is denoted as θ (in radian). Then the output current magnitude is calculated as (1a) and the simplified equations for cases where φ=0 and π∕6 are written in (1b) and (1c), respectively. Equation (1c) is used by the sparse-sampling-based power calculation in this paper.
(1) {iopk(k)⋅sin(θ+φ)=io(k)iopk(k)⋅sin(θ+π2)=io(k+1)}
(1a) $$i_{opk}\;(k)=\;\sqrt{\frac{|i_{o}\;(k)\;{|}^{2}+|i_{o}\;(k+1){|}^{2}-2\cdot |i_{o}\;(k)\;|\cdot |\;i_{o}\;(k+1)|\cdot \sin\;(\varphi )}{cos^{2}\;(\varphi )}}$$
(1b) $$i_{opk}\;(k)=\sqrt{|i_{o}\;(k)\;{|}^{2}+|i_{o}\;(k+1){|}^{2}}$$
(1c) $$i_{opk}\;(k)=\;\sqrt{\frac{4\cdot (|i_{o}\;(k)\;{|}^{2}+|i_{o}\;(k+1){|}^{2}-i_{o}\;(k)\;|\cdot \;i_{o}\;(k+1)|)}{3}}$$
4. Then, the load impedance magnitude is estimated as (2):
(2) $$|Z|=\frac{V_{o}\;(k+1)}{i_{opk}\;(k)}$$
5. The impedance angle θ is approximated by (3):
(3) $$\theta =\cos^{-1}\frac{|i_{o}\;(k+1)|}{i_{opk}\;(k)}$$
6. The estimated load impedance is captured in (4):
(4) Z=∣Z∣∠(θ)
7. Finally, the continuous instantaneous and average output power per cycle is obtained by multiplying output current and voltage, and then averaged over one cycle. They are dictated in (5) and (6), respectively:
(5) $$P_{o}\;(t)=\frac{V_{opk}\cdot I_{opk}}{2}\cdot \cos\;(\theta )-\frac{V_{opk}\cdot I_{opk}}{2}\;\;\cdot \cos\;(4\pi f_{s}t+\theta )$$
(6) $${\bar{P}}_{o}\;(t)=\frac{V_{opk}\cdot I_{opk}}{2}\cdot \cos\;(\theta )$$

where $V_{opk}$ equals to $V_{o}(k+1)$ and is the output voltage peak while $I_{opk}$ is the peak of output current and $f_{s}$ is the output frequency. With the assumption of sinusoidal output voltage and current, the proposed PCA can determine average output power and load impedance in a cycle-by-cycle ultrafast manner with only one voltage sample and two current samples, respectively. Hence, compared with existing literature, the ADC sampling rate requirement is significantly reduced. Furthermore, the PCA only involves simple mathematical operations with low complexity, which enables its real-time implementation in low-end industrial level DSP.

### B. Impact of Arcing Presence on Output Current and Power Calculation

**Fig. 4.**
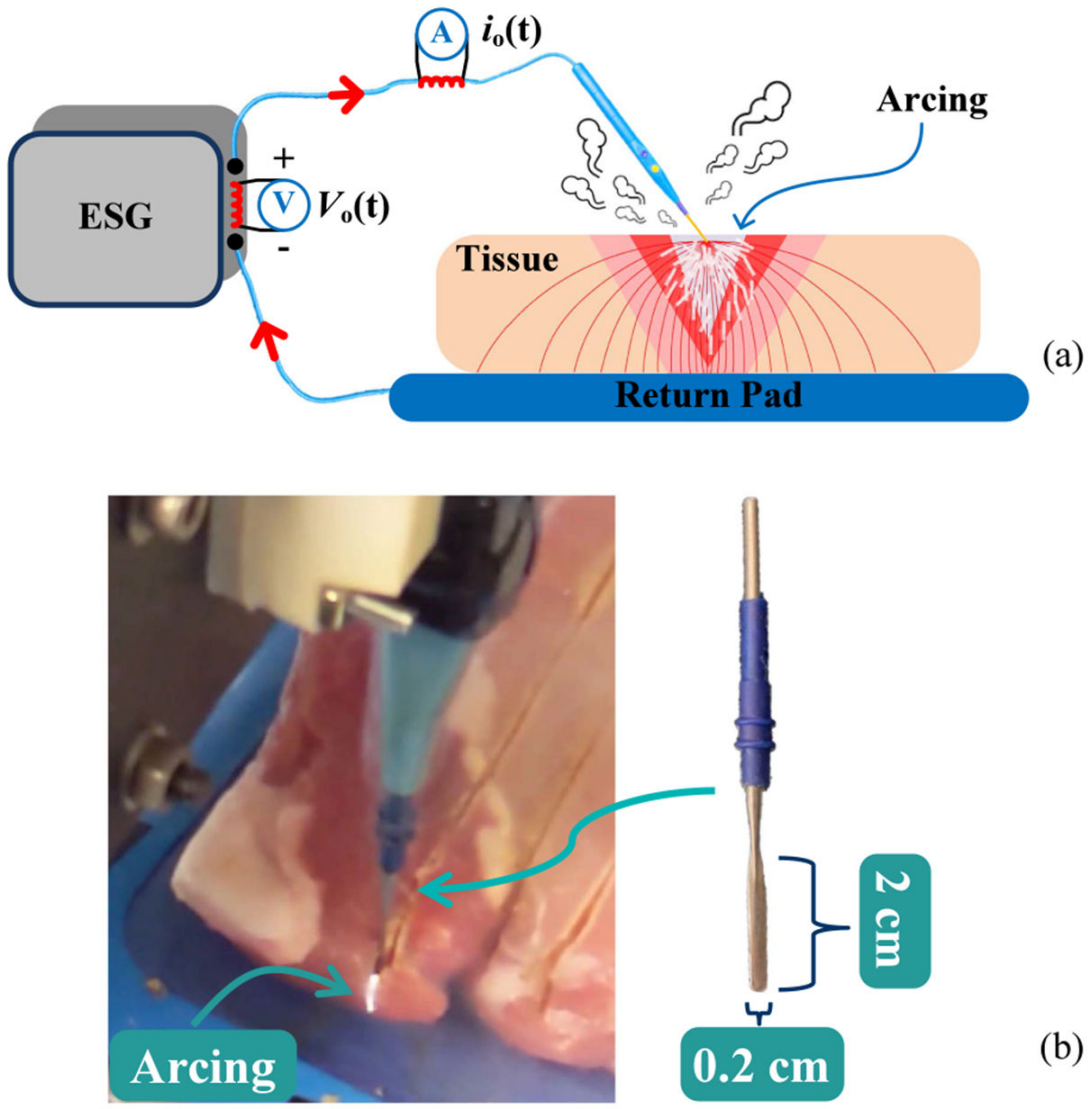
(a) Graphical representation of arcing during electrosurgery. (b) Experimental demonstration of arcing. The gauged dimension of the blade electrode used in this paper is marked on the right side.

It is worth mentioning that both instantaneous and average power computations using (5) and (6) assume a linear load. It means that the sinusoidal voltage induces sinusoidal current with the same frequency. This assumption is adopted by most of the existing literature, and it is true if no arcing is generated by the applied voltage during electrocuting, which is generally not the case in reality. On the opposite, actual electrosurgery is often accompanied by nonlinear arcing so that the current is no longer sinusoidal. Arcing occurs when the air around the electrode is broken down (ionization of dielectric [17]), as graphically represented in Fig. 4(a) while Fig. 4(b) experimentally demonstrates the occurrence of arcing during electrocuting on fresh pork. With the presence of arcing, the lumped currents seen by the electrode have two parts. One part is introduced by the tissue impedance along with the interface impedance between the electrode and tissue. The other part is due to arcing resistance, biotissue impedance, and interface impedance between them. The lumped current is distorted and deviates from a purely sinusoidal shape due to the nonlinearity introduced by the arcing [17], [18]. The degree of lumped current distortion largely depends on the proportion of current flowing in the form of arcing.

With appreciable current distortion and potential voltage asymmetry or distortion, neither output current nor voltage should be assumed as sinusoidal anymore [18]. Because of that, the average output power cannot be calculated by the way described in (6), otherwise, tangible power calculation errors are inevitably induced. On the other hand, it is quite important for an ESG to accurately compute the output power and then precisely track the given reference [8], [12]. For these reasons, a real-time multi-sampling-based power calculating method is set forth herein with the expectation of enhanced power calculating precision. The detailed illustrative explanation for it is presented in the following subsection.

### C. Multi-Sampling-Based Power Calculation

**Fig. 5.**
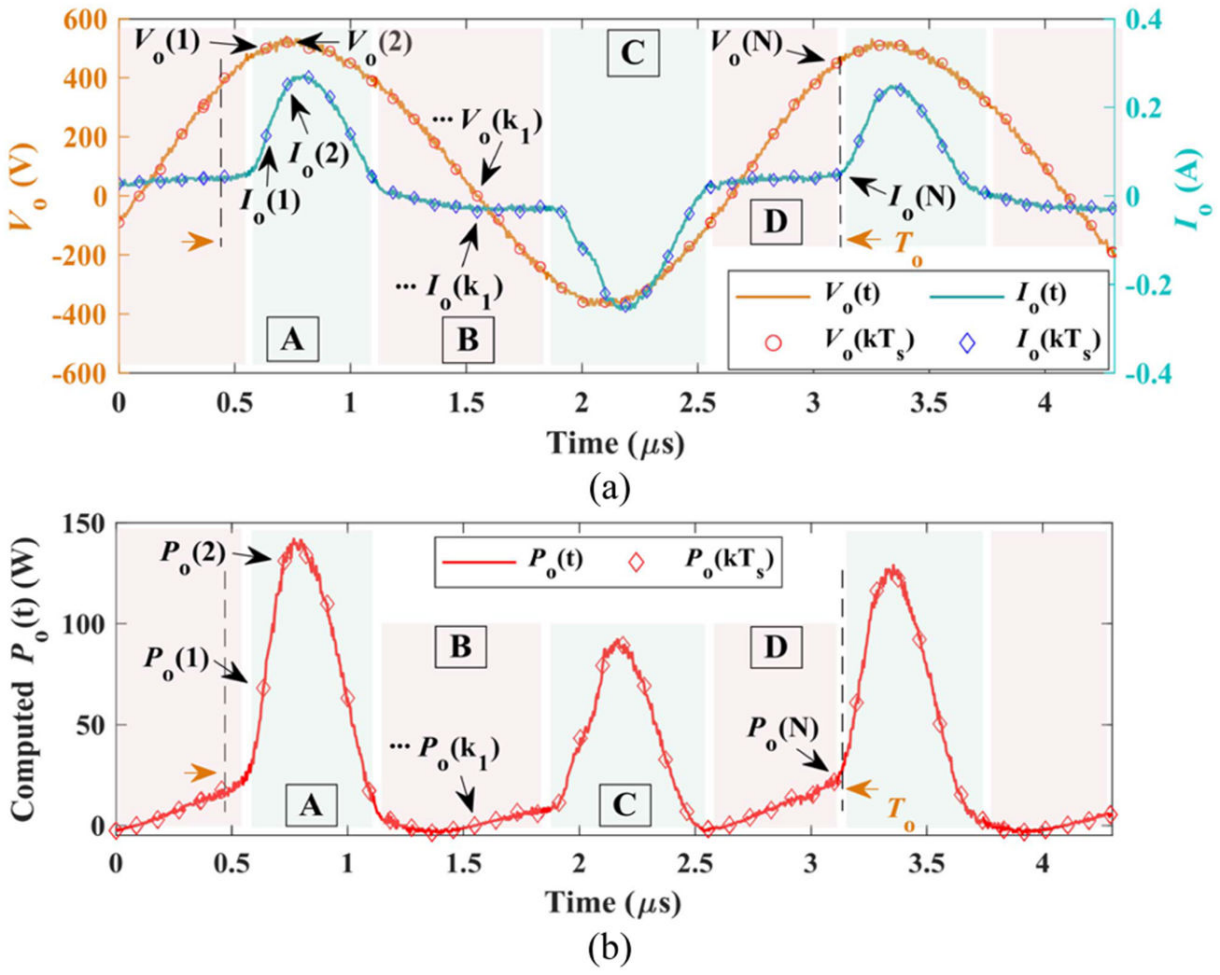
The multi-sampling-based average power calculation. (a) Output signals are sampled N times per output cycle T_o_. The ADC sampling period is denoted as T_s_. (b) The illustration of instant power. With 28 sampling points per cycle, the obtained mean value of power is 29.9 W for sampled discrete signals versus 30.2 W for original continuous one.

The presence of large arcing heavily distorts the output current, thereby, more sampling points are necessary to reflect current characteristics. A graphical representation of output voltage and distorted current is plotted in Fig. 5(a) with N samples each cycle. In Fig. 5(a), the output voltage and current are simultaneously sampled at the same timing as a pair and N pairs in total each cycle. Multiplying voltage and current, the obtained instant power is drawn in Fig. 5(b) for both original and sampled outputs. The instant power pulsates twice each cycle with nonidentical amplitudes and nonsinusoidal profiles, which highlights the inapplicability of (5) and the hypothesis of sinusoidal outputs again. The instantaneous power and average power for sampled outputs over one cycle are expressed by (7) and (8), respectively.

(7) $$P_{ins}\;(k)=V_{o}\;(k)\;\cdot \;I_{o}\;(k)$$

(8) $$\langle {\bar{P}}_{avg}\;(j)\rangle_{T_{o}}=\left(\sum_{k=(j-1)\cdot N+1}^{(j-1)\cdot N+N}P_{ins}\;(k)\right)\;∕N$$

where $V_{o}(k)$ and $I_{o}(k)$ symbolize $k_{th}$ instant value of output voltage and current in each cycle, respectively. k ranges from 1 to N. $P_{ins}(k)$ stands for the $k_{th}$ instant powe. $\langle {\bar{P}}_{avg}(j)\rangle_{T_{o}}$ represents the $j_{th}$ average output power over one output cycle $T_{o}$ (or over N instantaneous power samples).

In Fig. 5, the distorted output current is approximately divided into 4 linear regions, namely A-D. Compared to regions A and C, the output voltage is relatively small in regions B and D. Accordingly, less arcing are supposed to occur in region B and D, then the lumped load current is small and dominant by the tissue impedance and the interface impedance between tissue and the electrode. Owing to limited voltage and small load current, the yielded instantaneous power is also insignificant. However, more arcing is involved in regional A and C as applied voltage augments, therefore, the lumped load current rapidly surges, as depicted by the sharp current corner in Fig. 5(a). The instant powers in these regions have a substantial impact and predominate the average power over a cycle. Provided the dominant impact of regions A and C on overall averaged output power, the load impedance is defined as (9). Due to the similarity between regions A and C, only region A is selected to calculate the load impedance that is exploited later for power adaptation.

(9) $$|Z|\;(k)=max\;(V_{o}\;(kT_{s}))\;∕max\;(I_{o}\;(kT_{s}))$$

where ∣Z∣(k) is the load impedance while $max(V_{o}(kT_{s})$ and $max(I_{o}(kT_{s})$ represent the largest value of sampled output voltage and current within region A, respectively. If needed, then the average value of $\langle {\bar{P}}_{avg}\rangle_{M\cdot T_{o}}$ and $\langle |Z|\rangle_{M\cdot T_{o}}$ over M cycles can also be defined as (10) and (11).

(10) $$\langle {\bar{P}}_{avg}\rangle_{M\cdot T_{o}}=\left(\sum_{j=1}^{M}\sum_{k=(j-1)\cdot N+1}^{(j-1)\cdot N+N}V_{o}\;(k)\cdot I_{o}\;(k)\right)\;∕\;(M\cdot N)$$

(11) $$\langle |Z|\rangle_{M\cdot T_{o}}=\left(\sum_{k=1}^{M}|Z|\;(k)\right)\;∕\;M$$

where $\langle {\bar{P}}_{avg}\rangle_{M\cdot T_{o}}$ indicates the averaged output power over M output cycles (or all M⋅N instant power samples) while $\langle |Z\rangle_{M\cdot T_{o}}$ denotes averaged impedance over M cycles.

Viewing the maximum ADC sampling rate of DSP used for this paper, the value of N in (8) is selected as 28 herein. With 28 samples each cycle, the instant output powers are derived as an example for original continuous signals and discretely sampled data as exhibited in Fig. 5. The mean value of power is very close to each other with only 0.3 W arithmetic errors even with the presence of heavy current distortion. This error is conjectured to be much smaller than that using (5).

### D. Principle of Impedance-Based Power Adaptation

As stated before, the thermal-feedback-based power adaptive method can reduce collateral tissue damage, but its performance is affected by lots of factors. Therefore, impedance-based ultrafast power adaptation is proposed to conquer the influence of those factors. The principle of this novel methodology is thoroughly explained in this section.

**Fig. 6.**
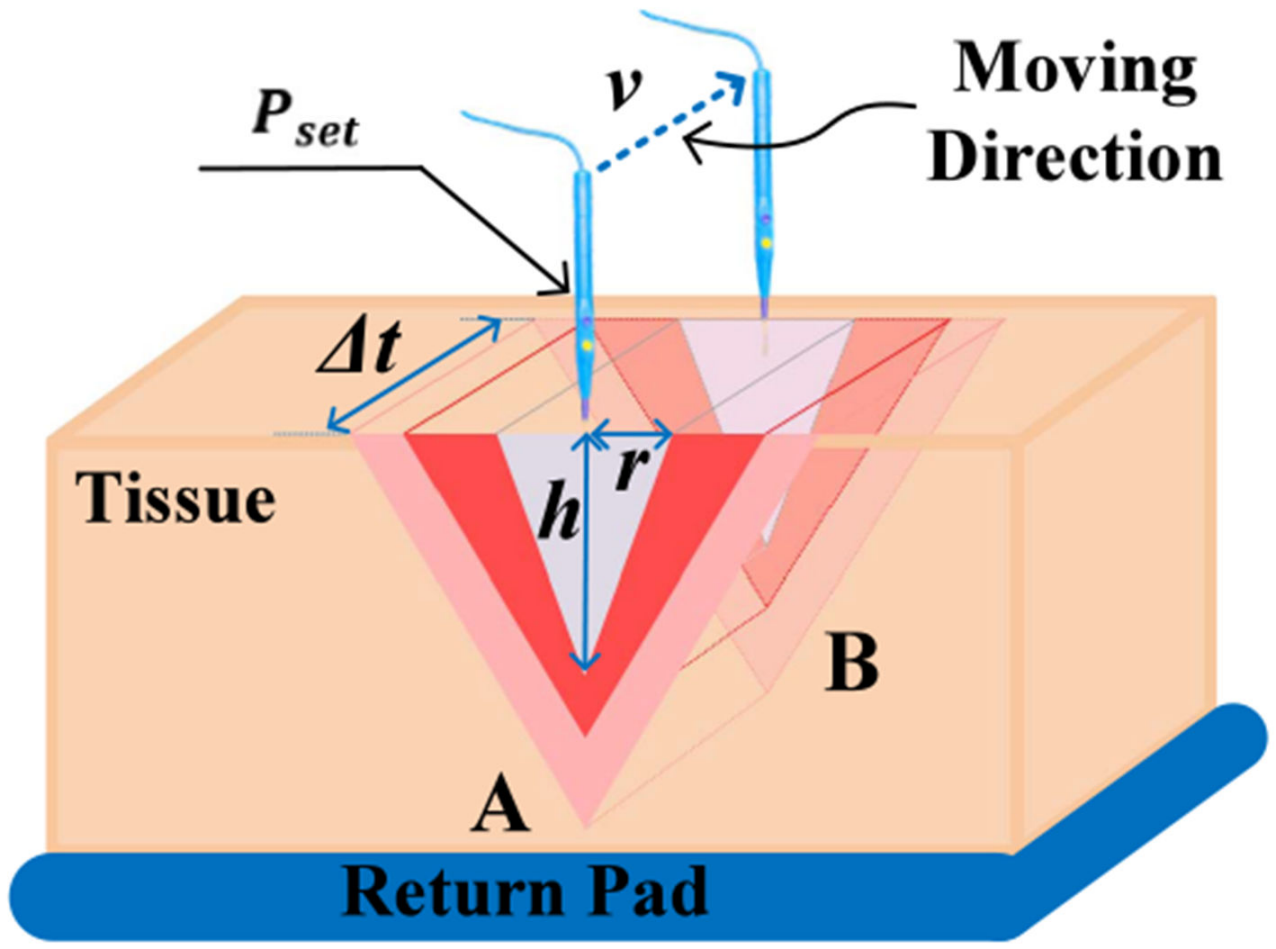
The illustration of electrocuting from point A to B with a triangular cross-section. The silver-gray layer represents tissue being removed and the red region designates tissues denaturized while the pink zone witnesses the overheated area. The electrode insertion depth is denoted as h (m) while cutting width and cutting speed are specified as r (m) and v (m/s), respectively. The electrocuting duration Δt illustrated here is extremely short.

It is reported that as the electrode designed for electrocuting gets in touch with the biotissue, the high-density current flows through the advancing edge of the tissue, followed by gradually decreased current density inside the tissue [3], [19]. The temperature profile of the tissue during electrocuting also quickly drops down as the radial distance measured from the electrode increases [11]. Consequently, only tissue within a few millimeters (mm) radial distance from the electrode is vaporized and removed in the hypothetical shape of an inverted cone. Fig. 4(a) illustrates the cross-section of such an inverted cone in a silver-gray triangle, and it is redrawn below in Fig. 6 plus many other notations. With the information offered, the ideal energy that induces zero collateral damage when the electrode moves from point A to B is given by (11a):

(11a) $$P_{idl}\cdot \Delta t=m\cdot c_{eq}\cdot \Delta T=\frac{1}{2}\cdot r\cdot h\cdot v\cdot \Delta t\cdot \rho \cdot c_{eq}\cdot \Delta T$$

where $P_{idl}$ (W) is the ideal average output power over one cycle, Δt (s) is the cutting time duration and m (kg) is the mass of the targeted tissue. ΔT (K) is the temperature rise while $c_{eq}$ (J ·kg^−1^ · K^−1^) and ρ (kg/m^3^) are the equivalent specific heat capacity and density of the tissue, respectively. They can be approximately processed as a constant for tissues of similar constituents without causing too many errors.

To simplify analyses, 3 conditions are assumed as follows:

1. The cutting radial distance r is generally quite small [11] and can be assumed as a fixed value.
2. The temperature discrepancies ΔT from normal tissue status to the vaporizing point are fixed, such as from normal body temperature of 37 °C to 100 °C.
3. The load impedance magnitude during electrocuting is inversely proportional to the product of electrode insertion depth and moving speed, as depicted by (12a). The (12b) and (12c) are derived when one of the variables in (12a) is fixed.

(12a) $$|Z|=\lambda \cdot \frac{1}{h\cdot v}$$

(12b) $$|Z|=\alpha \cdot \frac{1}{h}$$

(12c) $$|Z|=\beta \cdot \frac{1}{v}$$

where λ is the coefficient that relates the ∣Z∣ with the h⋅v (m^2^/s). α and β is the coefficient bridging the load impedance magnitude ∣Z∣ with electrode insertion depth h(m) and moving speed v (m/s), respectively. Instead of function type, different tissue types differ from each other only by separate coefficient values in (12). Condition 3) is experimentally certified for muscle tissue and documented in Section III-E.

Plugging (12a) into (11a), (13) is elicited as follows:

(13) $$P_{idl}\cdot |Z|=\gamma$$

where γ is a constant and equates to $\frac{1}{2}\cdot r\cdot \lambda \cdot \rho \cdot c_{eq}\cdot \Delta T$.

**Fig. 7.**
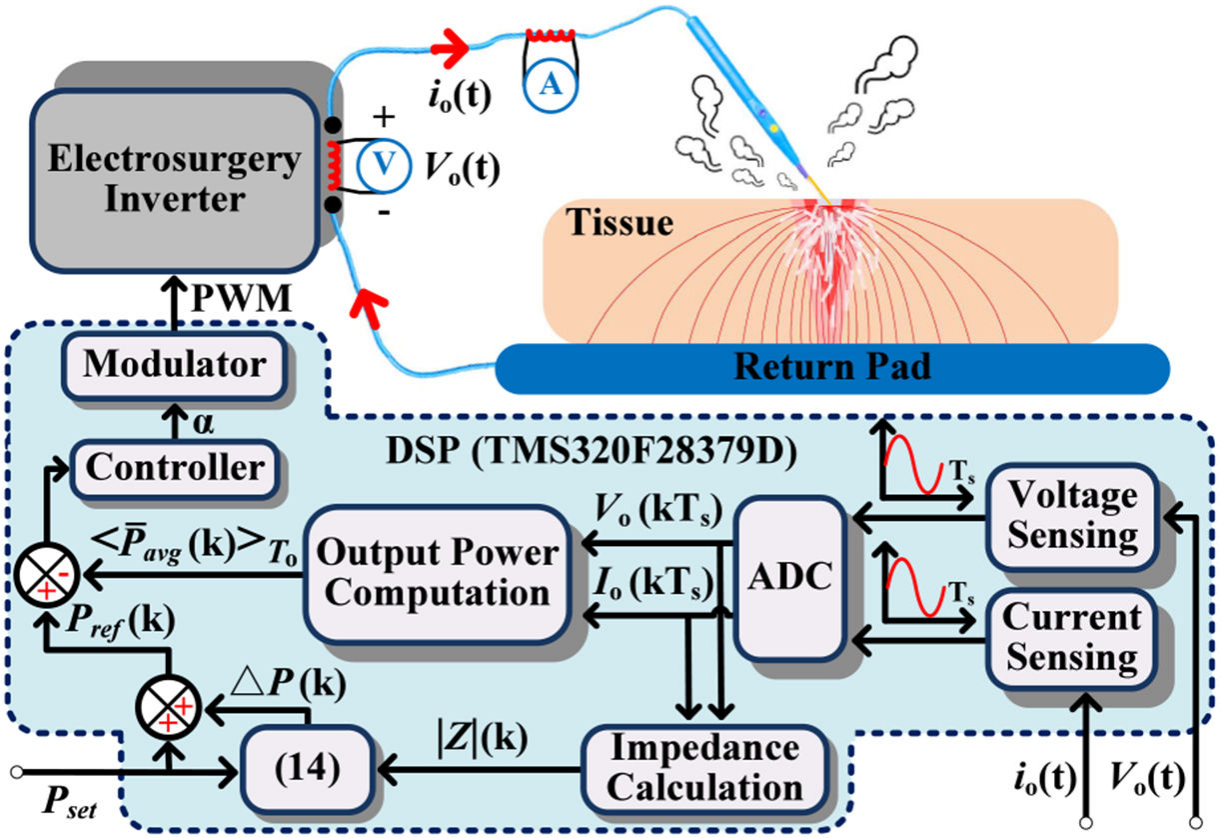
Closed-loop control block diagram. The modulator outputs pulse-width modulation (PWM) signals that switch GaN devices in electrosurgery inverter.

From (13), the multiplication of ideal power and load impedance magnitude is a constant. To ensure $P_{ref}(t)$ is equal to $P_{idl}$ for reduced damage as load impedance varies, the real-time power adaptation can be generated as (14) and added to preset power reference $P_{set}$ from surgeons, yielding (15):

(14) $$\Delta P\;(t)=\gamma \cdot \left(\frac{|Z_{0}|-|Z|}{|Z|\cdot |Z_{0}|}\right)$$

(15) $$P_{ref}\;(t)=P_{set}+\Delta P\;(t)$$

where $|Z_{0}|$ is equal to $\gamma ∕P_{set}$. Based on (14) and (15), the impedance-based power adaptation strategy with reduced collateral tissue damage from Fig. 4(a) is delineated in Fig. 7 together with controller output α and modulator outputs.

## III. Results

### A. Sparse-Sampling-Based Power Calculation

**Fig. 8.**
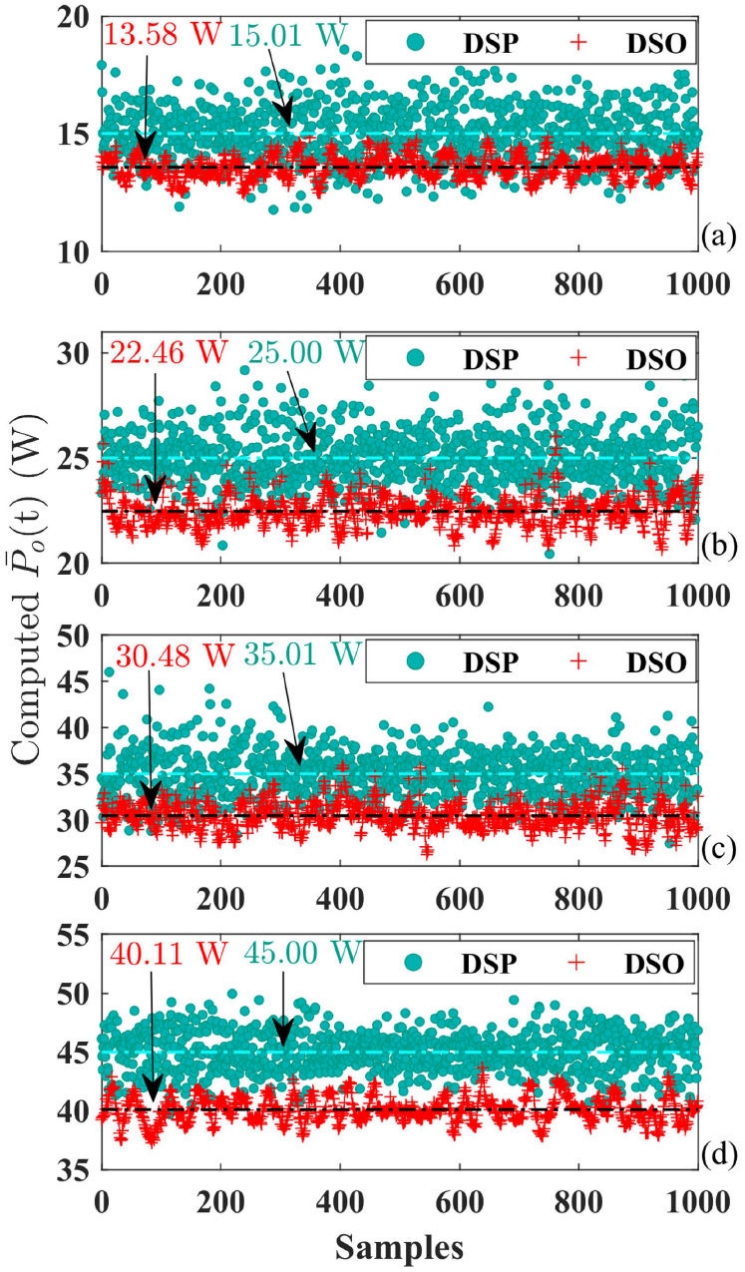
Accuracy examination of sparse-sampling-based power calculation. The power value in the digital signal processor is denoted as DSP while those produced by data from the digital storage oscilloscope is marked as DSO. The DSP used here is TMS320F28379D. The mean value over all 1000 samples is designated by the colorful texts. (a) 15 W. (b) 25 W. (c) 35 W. (d) 45 W.

The accuracy of power yielded from the sparse-sampling-based algorithm is experimentally examined by cutting fresh pork using the HFI introduced in [13]. The output power of HFI is under closed-loop control and the power reference in Fig. 8(a)-(d) ranges from 15 W to 45 W with a step of 10 W. The averaged output powers obtained using (6) are partially extracted from DSP for 1000 cycles and displayed in Fig. 8 together with those from the digital storage oscilloscope (DSO) for different power settings. The data from DSO is sampled at 200 MS/s with sufficient long recording length and then average power is mathematically computed on the basis of those massive data. Therefore, the results from DSO are treated as the most precise reference herein. As concluded from Fig. 8(a)-(d), the mean value over all 1000 cycles for DSP and DSP are very close to each other, however, the deviation escalates as the delivered power strengthens. On top of that, instant power in DSP deviates from the average value with relatively large errors for part of the cycles. In another word, the sparse-sampling-based PCA features fairly accurate power computation for most of the cycles, and only a small proportion of samples need improvement.

### B. Impact of Arcing Presence on Output Current

**Fig. 9.**
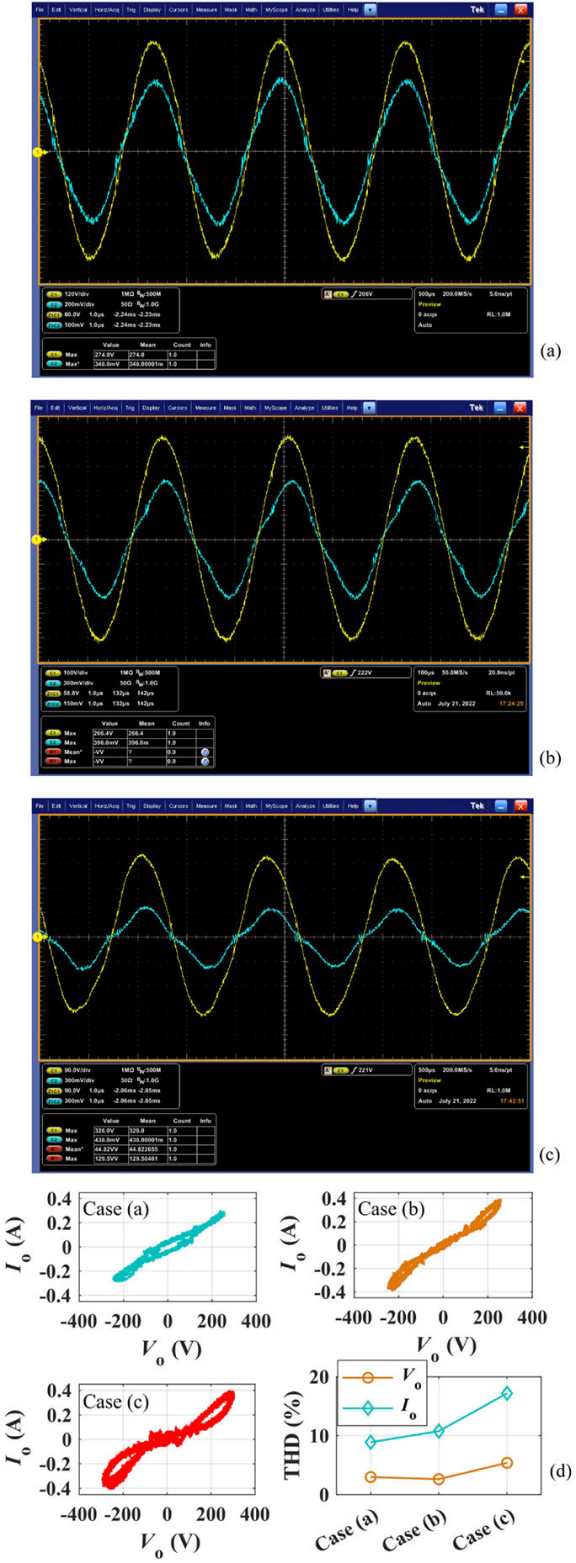
Illustration of output current (cyan trace) distortion present during electrocuting: (a) barely distorted, (b) slightly distorted, and (c) appreciably distorted. Among the two traces, the yellow one with higher magnitude is the output voltage. (d) Output current versus voltage together with their THD.

The impact of arcing on output current distortion can be qualitatively probed through revising the output voltage or insertion depth, etc. Fig. 9(a)-(c) experimentally demonstrate different degrees of current distortion during electrocuting. Output current versus voltage is plotted in Fig. 9(d) for all of them together with the total harmonic distortion (THD). As shown in Fig. 9(d), for cases (a)-(c) in Fig. 9(d), the arcing hysteresis becomes more and more visible as the current distortion escalates. Meanwhile, the current THD is also rapidly aggrandized as manifested by the subfigure in the bottom right corner of Fig. 9(d). Nevertheless, the output voltage THD consistently remains at a low level due to the specifically designed filter elaborated in [13].

### C. Multi-Sampling-Based Power Calculation

**Fig. 10.**
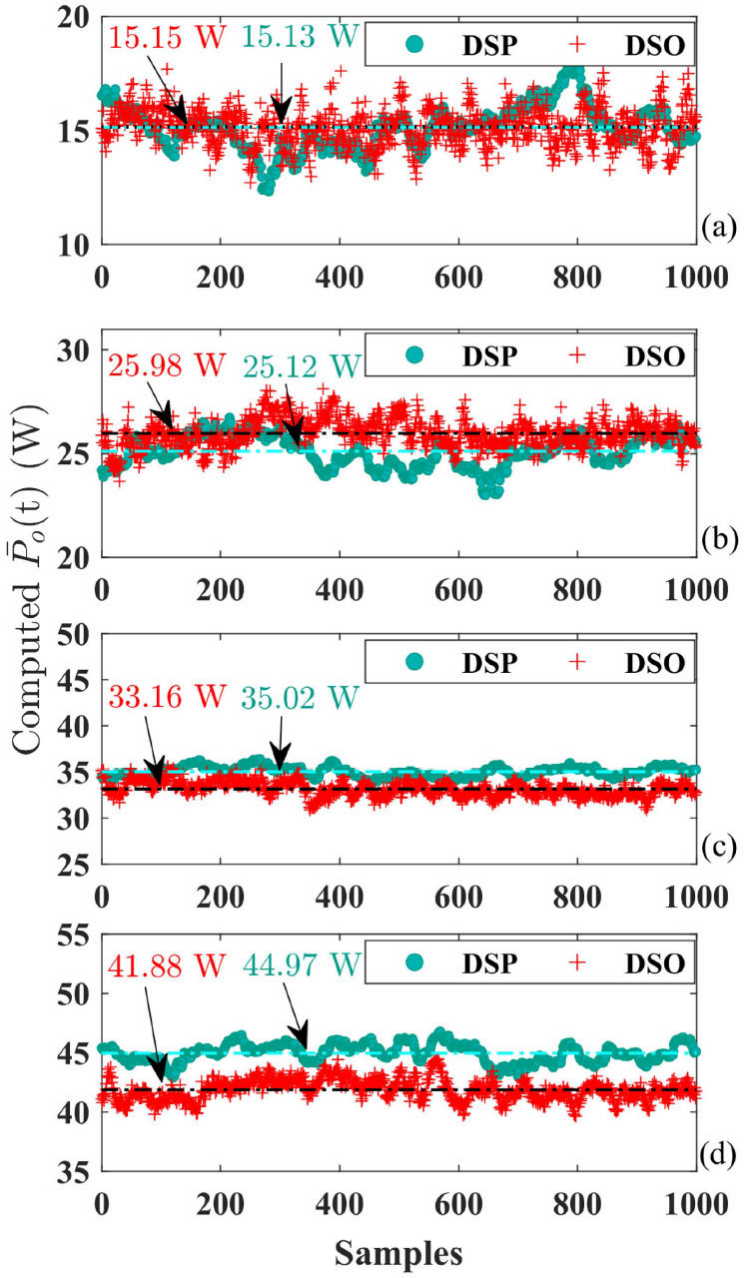
Power accuracy test of multi-sampling-based method. The values from DSP or DSO are separated by legend. (a) 15 W. (b) 25 W. (c) 35 W. (d) 45 W.

The computing accuracy performance of the new power quantification method is experimentally checked under the same experimental settings for the sparse-sampling-based approach. The acquired average power from the DSP and those got from DSO data are revealed in Fig. 10. Compared with results in Fig. 8, the power values produced by the multi-sampling-based method cluster much closer to the actual values from the DSO. Furthermore, the mean value of power over all 1000 samples from the new method is more accurate with fewer deviation errors than that in Fig. 8. The comparison between power computing accuracy in Figs. 8 and 10 is summarized in Table I and affirms the necessity of employment of the multi-sampling-based power gauging method if lower tolerance is preferred.

### D. Steady-State Power Tracking Performance

**Fig. 11.**
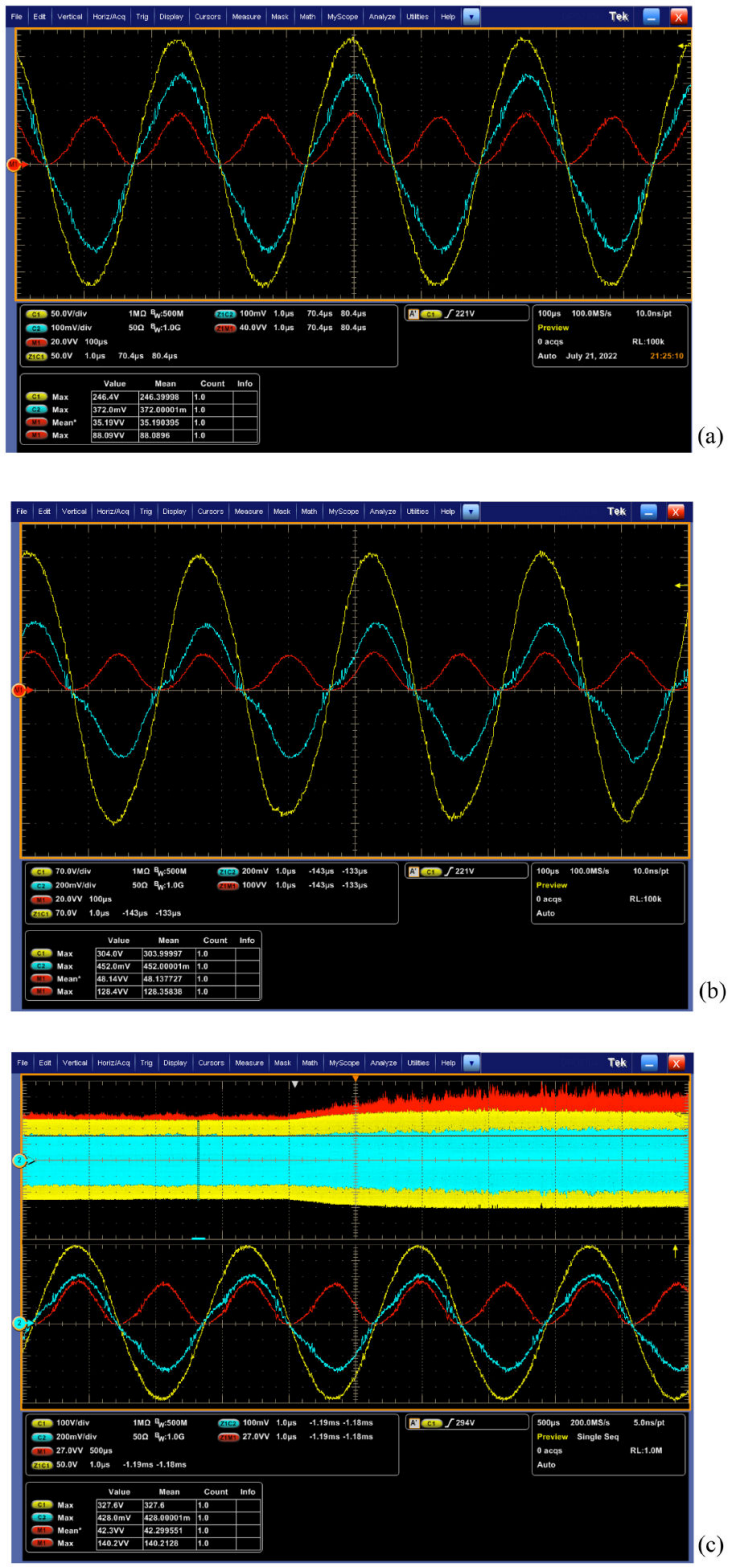
Steady-state power tracking performance: (a) 35 W and (b) 50 W. (c) Power steps from 35 W to 50 W. The output power is the trace in red while the cyan and yellow trace represents output current and voltage, respectively.

Utilizing the new power computing method, the steady-state power tracking performance of HFI in [13] is investigated with a proportional-integral controller and showcased in Fig. 11. In Fig. 11(a), DSO manifests a 35.2 W power averaged over all cycles when the power tracking reference is set as 35 W in DSP. Fig. 11(b) implies a 48.1 W steady-state power in DSO when the system is tracking 50 W. Combining Fig 11(a) and (b), it can be declared that the system tracks the preset power reference with tiny errors. Fig. 11(c) captures the system transitional dynamics, where the power reference is initially set as 35 W, and then, directly shifted toward 50 W.

### E. Relationships of Load Impedance Against the Electrode Insertion Depth and Cutting Speed

**Fig. 12.**
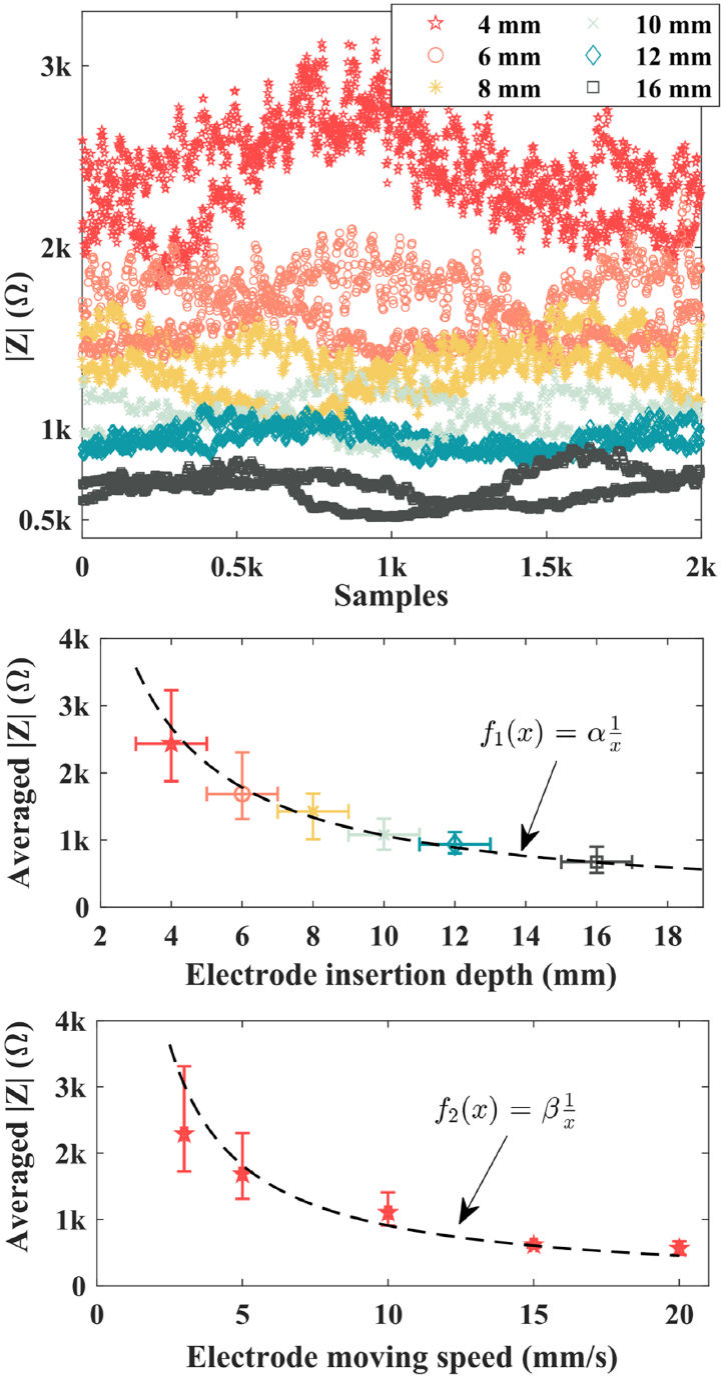
Experiment verification of condition 3). The insertion depth has the unit of millimeter (mm) here and the electrode moving speed is denoted in mm/s. And then, parametric value of λ in (12) is around 54,000 while parameters α and β are equal to 10,700 and 9,100, respectively. However, the value of λ is reduced to 0.054 if the h⋅v is in the unit of m^2^/s.

To authenticate condition 3) mentioned in Section II-D, electrocuting trials with various insertion depths and cutting speeds are conducted with the help of a programmable Emile3 3-axes robotic gantry. Experimental results are documented in Fig. 12. The top subfigure in Fig. 12 presents the secured load impedance defined in (9) versus the electrode insertion depth. In the middle subplot, electrode insertion depth varies from 4 mm to 16 mm with a ± 1 mm error bar, and the midpoint of load impedance over 2000 samples is graphed on the vertical axis. It is observed that the earned load impedance versus electrode insertion depth h approximately fits an inverse proportional function $f_{1}(x)$. In the bottom subplot, the load impedance is plotted versus the electrode moving speed V, and the curve roughly fits the inverse proportional function $F_{2}(X)$ if the cutting speed is not too slow. Combining both functions $f_{1}(x)$ and $f_{2}(x)$, the load impedance is deduced as an inverse proportional function of h⋅v that is in the form of (12a). Therefore, the experiment results in Fig. 12 advocate the validity of condition 3) in Section II-D.

### F. Impedance-Based Power Adaptation

By following (13), the power reference is so adjusted such that the product of $P_{ref}(t)$ and ∣Z∣ is brought back to γ as ∣Z∣ varies. In this way, the actual electrocuting is near to ideal cutting with minimal collateral tissue damage. It is noteworthy that the exact value of γ is hard to be theoretically calculated, therefore, its approximated optimal value is figured out by extensive experiments. Based upon massive trials, the value of γ used in this paper is 30000, and all load impedance in DSP is smoothed out by the approach of moving average. The process of moving averaging enhances system power tracking stability by eliminating power reference violent fluctuation that originates from load impedance sudden jump. The larger the moving average window length, the better tracking stability. However, it also compromises prompt power adaptation dynamics by slowing it down. To keep a balance, the load impedance in this paper is monitored each cycle, but all impedance values used in (14) for the power reference regulation are their moving average scrolling over 10 cycles. Based on that, the efficacy of the load impedance-based power adaptation method is scrutinized with multi-sampling-based output power computation in Fig. 7, and 5 test scenarios are tabulated and listed in Table II.

In test scenario 1, the cutting speed is 5 mm/s and $P_{set}$ power is kept the same at 35 W during the entire electrocuting. The electrode is fixed on the programmable Emile3 3-axes robotic gantry and its moving speed is under control for the sake of experimental repeatability. Instead of constant power configuration, test scenarios 2-5 are equipped with power adaptation. And their cutting speeds along with electrode insertion depth are intentionally configured differently to examine the general applicability of the proposed impedance-based power adaptation philosophy. The purpose of test scenario 1 is to emulate conventional electrosurgery and it also provides a reference criterion of collateral tissue damage for test scenarios 2-5 as explained later in Table III.

**Fig. 13.**
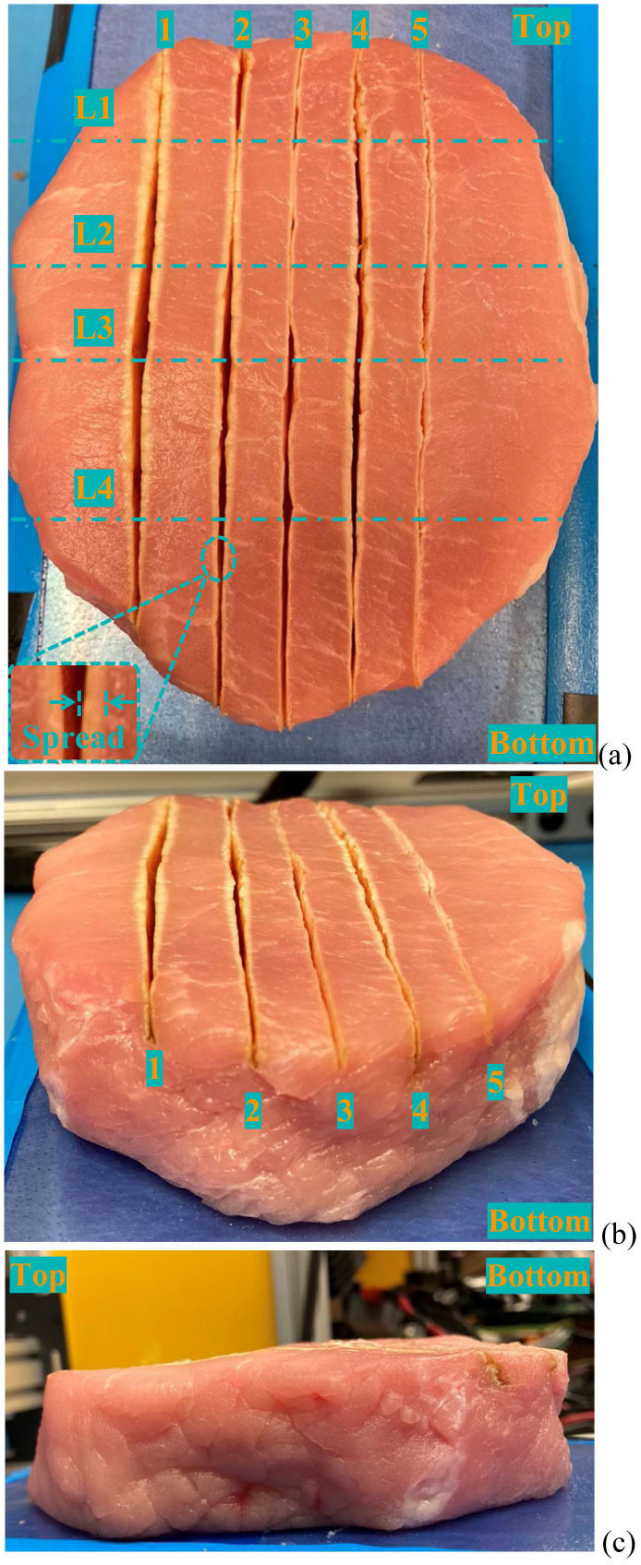
Pictorial capture of 5 electrocuting traces. (a) Top view. (b) Front view. (c) Left-side view.

Following the experimental setting listed in Table II, fresh pork is cut from the top to bottom, and 5 electrocuting traces are outcomes, as present in Fig. 13. From Fig. 13(c), it is seen that the biotissue surface is not flat, and it is often the case in actual electrosurgery. Thus, it is reasonable to vary the electrode insertion depth with a noticeable step to investigate the performance of the proposed impedance-based power adaptive approach. The quality of cutting trace is evaluated in terms of thermal spread which is exemplified in Fig. 13(a). The thermal spreads of all 5 cutting traces are metered at 4 cross points with lines 1-4, namely, L1-L4. The narrower the thermal spread, the better the cutting quality since less thermal spread signifies reduced collateral tissue damage. The gauged thermal spread is summarized in Table III. As noticed in Table III, test scenario 1 conducted with constant power has maximum thermal spread. With the impedance-based power adaptation, the thermal spread of test scenario 2 is significantly reduced from test scenario 1. When the electrode insertion depth exceeds more than half of the entire electrode blade, as in scenario 4, the power adaptation can still reduce thermal spread, but the reduction is not as much as that in a shallow case much like test scenario 2. But in actual electrosurgery, the electrode insertion depth is controlled by surgeons with a routinely shallow insertion depth (around 6 mm) since deep cutting can be achieved by several shallow slices. Looking through test scenarios 2 and 3 or test scenarios 4 and 5, it is verified that the proposed impedance-based power adaptation works well even if cutting speed varies. For test scenarios 2 and 4 (or test scenarios 3 and 5), the cutting speed is maintained at 5 mm/s (or 10 mm/s) while the electrode insertion depth differs. It is seen that the thermal spread is always smaller for traces cut at 10 mm/s than that at 5 mm/s. It underlines the important role of cutting speed in reducing thermal spread once more. Aside from that, it might also forecast the necessity to massively collect habitual cutting speeds preferred by surgeons such that the value of γ is optimized for reductive thermal spread.

**Fig. 14.**
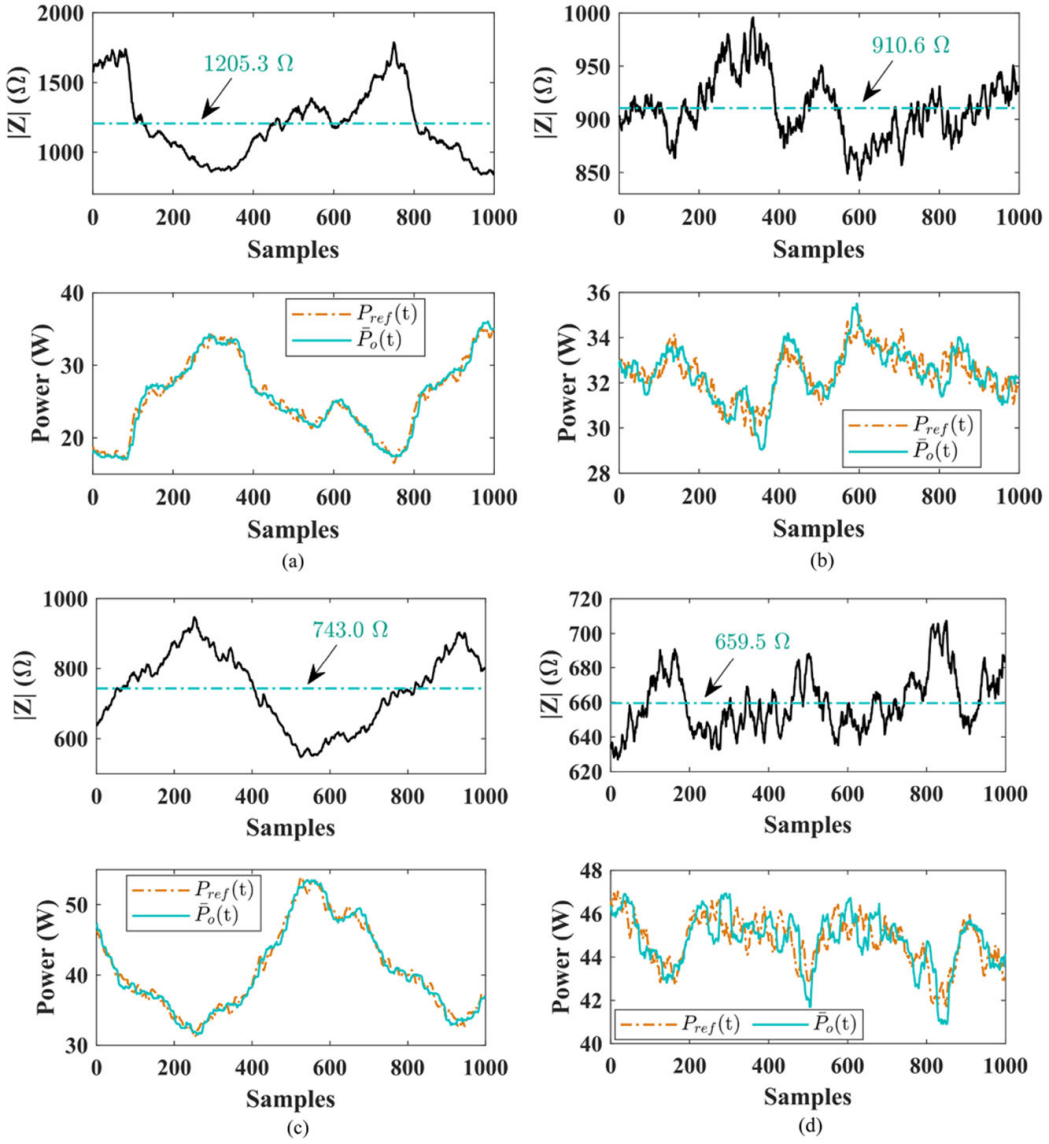
Fragment of load impedance along with power reference $P_{ref}(t)$ and average output power ${\bar{P}}_{o}(t)$ per cycle. The mean value of load impedance over all 1000 samples is also specified by the text. (a) Captured from trace 2. (b) Captured from trace 3. (c) Captured from trace 4. (d) Captured from trace 5.

As seen in (14) and (15), the proposed impedance-based power adaptation method supervises the load impedance in real time and adjusts the power reference cycle by cycle. It implies that power reference updates in a few microseconds in consideration of 390 kHz output frequency. It is much faster than the thermal-based power adaptive manner. Fragment of power references during electrocuting together with measured load impedance for test scenarios 2-5 are plotted in Fig. 14(a)-(d), respectively. In Fig. 14, the power reference $P_{ref}(t)$ is finely tuned per load impedance and it is also closely followed by system average output power ${\bar{P}}_{o}(t)$. Thereby, it proves the feasibility of the impedance-based power adaptation technique and also validates the ultrafast power tracking capability of the entire high-frequency inverter system.

## IV. Discussion

The article briefly illustrates the current paths inside the biotissue and elucidates the electrocuting mechanism to pave the way for a basic understanding of electrosurgery. Conventional electrosurgery delivers constant power to the target tissue with high accuracy. It increases the possibility of added collateral tissue damage. Surgeons can frequently and manually modify power settings to better cutting quality with less tissue damage. At the cost of doing that, the termination of time-sensitive electrosurgical processes is unavoidable and lengthens the clinical duration which might lead to serious consequences or cause potential exposure to a higher risk of clinical surgical failure. Therefore, autonomous real-time power adaptation is of paramount importance.

With the information on tissue surface temperature, the thermal-feedback-based power adaptation can reduce tissue collateral damage during electrosurgery. But it requires one extra thermal sensor and resultant cost, heavy thermal data processing, fast communication, etc. Besides that, it is also experimentally revealed in this paper that the accuracy of temperature measurement is notably affected by thermal sensor mounting locations. Moreover, thermal sensor resolution and the existence of smoke during electrosurgery impose an impact on the thermal measurement precision as well. Because of that, either a costly thermal sensor with good resolution or a smoke evacuation pencil is needed to get rid of those impacts. Additionally, an advanced thermal sensing compensating algorithm might be another alternative to tackle the smoke issue for thermal sensing. Howbeit, with the constraint of thermal sensor refresh rate, the thermal-based power adaptation can hardly refresh the power reference beyond 100 Hz, which sets a barrier to its application in circumstances requiring ultrafast power adjustments. To crack all limitations or downsides related to the thermal-based approach, the load impedance-based power adaptation method is brought forward. Before the detailed explanation of such method, two schemes to calculate average output power with limited output measurements are formulated.

The sparse-sampling-based method requires the assumption of sinusoidal output voltage and current, which is not always true for actual electrosurgery due to the presence of arcing. But it dramatically shrinks the sampling requirements and calculates output power with only one voltage sample and two current samples each cycle. The power calculation results are analyzed through comparison with those from the digital storage oscilloscope which are thought as the most accurate. It turns out that the sparse-sampling-based method has satisfied power computing accuracy and only a small proportion of points are of relatively large errors. Therefore, the sparse-sampling-based tactic is suited for the case with limited processor computing power or with low accuracy concerns. It might need improvement for the application that is sensitive to power precision. This paper experimentally evidences the presence of arcing during electrocuting and demonstrates the current distortion. The distortion largely depends on the amount of arcing and the output current is no longer sinusoidal when heavily distorted. This observation accounts for relatively large calculation errors seen in Fig. 8.

Instead of sparse samples, the multi-sampling-based approach divides one output cycle into 4 linear regions for load impedance definition and calculates the output power with 28 samples per cycle. Experimental results show that the multi-sampling-based power computation values cluster very close to the values from the digital storage oscilloscope and have better power counting fidelity than that of the sparse-sampling-based formula. However, Table I reveals that power calculation errors still remain for the multi-sampling-based method with 28 samples of both outputs. As is well-known, the greater the sample number N per cycle, the more accurate the power calculation. Therefore, a sampling number N larger than 28 is needed to further reduce power computing errors. But larger N requires a higher sampling rate that is limited by the maximum achievable sampling speed on the ADC. Moreover, the computation burden also rapidly escalates as the sampling rate increases. The output frequency herein is 390 kHz with a rough period of 2.5 *μ*s, therefore, the digital computation speed is another key factor. In consequence, a tradeoff among power calculating accuracy, digital computing burden, and speed restriction should be maintained for satisfactory performance. Furthermore, the cycle number M in (10) poses an impact on power tracking performance in which a smaller M features prompt power tracking, but it also comprises system control stability. On the other side, an overlarge M ensures system stability whereas it also jeopardizes system tracking dynamics. And thus, the value of M should be properly selected to achieve a balance between prompt power response and tracking stability. In viewing of typical availability of maximum ADC sampling rate and ADC channels from low-end industrial-scale DSPs, the output current is sampled 14 times each cycle by two ADC channels in this paper. The sampling initiation point of each channel is so arranged such that all 28 sampling points are evenly distributed in time without overlapping. The less distorted output voltage is sampled 14 times each cycle and another 14 sampling points are interpolated between two actual samples. In other words, N in (8) equates to 28 in this paper. If more sampling points are crucial for ultrahigh power computation precision, then a dedicated data acquisition system or high-end FPGA with a very high ADC sampling rate is an alternative solution. With the rapid advancement of technology, normal digital processors might become sufficiently powerful to handle this task in the near future. Nevertheless, no matter now or future, the multi-sampling-based approach is more suitable for circumstances that have sufficient digital computing capability and need high fidelity as well.

Give the characteristics of two methods, it really depends on the actual situation to decide which way is better for power counting. In this paper, the way of multi-sampling is embraced by viewing the importance of power fidelity in electrosurgery.

Based on the definition of impedance, the relationships of load impedance against electrode insertion depth and cutting speed are established, followed by experimental proofs. Both correlations fit in the form of inverse proportional function. But it should be noted that load impedance against the electrode cutting speed seems to saturate and diverge from the inverse proportional form when the speed is too small. To that end, it might be necessary to make surveys and extensively gather the typically preferred cutting speed together with electrode insertion depth among surgeons. By doing so, it simplifies the problems, and then more accurate links among impedance, insertion depth, and cutting speed can be refined. In this paper, muscle tissue is used and its parametric properties, such as mass density, etc. are assumed to be uniform and there is no local variation. Even in this framework, it is still quite challenging to theoretically quantify the precise value of γ in (13) since the biotissue specific heat capacity is reported as a function temperature in the literature. On that account, it is also challenging to determine the value of equivalent specific heat capacity $c_{eq}$. Before $c_{eq}$ can be precisely determined, there is a need of extensive clinical trials to experimentally determine the approximated optimal value of γ for different tissue types. On the foundation of relationships just built, the impedance-based power adaptation is developed. This novel approach takes the thermal sensor away and updates the power reference in a cycle-by-cycle ultrafast manner, namely, in less than 3 *μ*s in this paper. It is orders of magnitude faster than the thermal-feedback-based power adaptation. Therefore, the proposed method is extremely suitable for cases that are very sensitive to power mismatch and need ultrafast power modulations, such as electrosurgery on the brain, cerebral vessels, heart, etc. If rapid power adaptation is not required, then it is also easy to slow down the power reference update rate by reducing load impedance monitoring frequency.

## V. Conclusion

This paper details and evaluates two novel ways of computing output power using limited output measurements for electrosurgery. The sparse-sampling-based method only samples output voltage once and current twice whereas it yields output power computation with small deviation errors. With slightly increased sampling points and computation burden, the multi-sampling-based method improves accuracy even when nonlinearity from electrosurgical arcing is present on the outputs. These two methods are implemented in low-end industrial-scale processors with limited sampling speed. Consequently, they may reduce the need for high-end processors with fast sampling speed and save corresponding costs for applications involving high-frequency and highly distorted outputs. Besides that, a mathematical relationship among defined load impedance, electrode insertion depth, and cutting speed is developed from the multi-sampling-based methodology. Evolving from the relationship, an original impedance-based power adaptation strategy is further formulated with the capability of autonomous power reference updating in a cycle-by-cycle ultrafast manner. Experiment results of different test scenarios show that electrocuting traces delivered by the impedance-based power adaptation strategy yield diminished thermal spreads from conventional electrosurgery, which validates the feasibility and efficacy of the impedance-based power adaptation strategy. Moreover, this power adaptation strategy eliminates the thermal sensor and eradicates the drawbacks associated with thermal-based power adaptation. In other words, collateral tissue damage in terms of thermal spread is reduced with the extra benefits of less sensor count and decreased cost. This demonstrates the superiority of impedance-based power adaptation over existing thermal-based power adaptation strategy. With all merits mentioned above, this work may stimulate more interdisciplinary research in the electrosurgical area and promote the development of zero-collateral-damage electrosurgery.

## Figures and Tables

**TABLE I:** Power Computation Accuracy Comparison

_Method_╲^Deviations^	Power Reference			
	@15 W	@25 W	@35 W	@45 W
Sparse-sampling-based	1.43 W	2.54 W	4.53 W	4.89 W
Multi-sampling-based	0.02 W	0.86 W	1.86 W	3.09 W

**TABLE II:** Configurations for 5 Test Scenarios

_Settings_╲^Trials^	Test Scenarios				
	#1	#2	#3	#4	#5
$P_{set}$	35 W	35 W	35 W	35 W	35 W
Cutting Speed	5 mm/s	5 mm/s	10 mm/s	5 mm/s	10 mm/s
Insertion Depth	Shallow	Shallow	Shallow	Deep	Deep
ΔP(t)	0 W	Using (18)	Using (18)	Using (18)	Using (18)
$P_{ref}(t)$	35 W	Using (19)	Using (19)	Using (19)	Using (19)

**TABLE III:** Evaluation of Electrocuting Quality

_Metrics_╲^Trials^	Test Scenarios				
	#1	#2	#3	#4	#5
Spread at L1	2.18	0.75	0.48	1.00	0.32
Spread at L2	1.63	1.27	0.35	1.10	0.34
Spread at L3	0.97	0.48	0.55	1.25	0.42
Spread at L4	1.20	0.96	0.37	1.12	0.39
Averaged Spread	1.49	0.86	0.43	1.11	0.36

Note: All measurements are gauged in millimeters (mm).
